# A putative AGO protein, OsAGO17, positively regulates grain size and grain weight through OsmiR397b in rice

**DOI:** 10.1111/pbi.13256

**Published:** 2019-10-07

**Authors:** Jun Zhong, Weijie He, Zhu Peng, Hui Zhang, Feng Li, Jialing Yao

**Affiliations:** ^1^ College of Life Science and Technology Huazhong Agricultural University Wuhan China; ^2^ College of Life Science Nanjing Normal University Nanjing China; ^3^ College of Horticulture and Forestry Sciences Huazhong Agricultural University Wuhan China

**Keywords:** OsAGO17, grain size, grain weight, cell elongation, OsmiR397b, rice

## Abstract

Argonaute (AGO) proteins and small RNAs (sRNAs) are core components of the RNA‐induced silencing complex (RISC). It has been reported that miRNAs regulate plant height and grain size in rice, but which AGO is involved in grain size regulation remains unclear. Here, we report that enhanced expression of *OsAGO17*, a putative AGO protein, could improve grain size and weight and promote stem development in rice. Cytological evidence showed that these effects are mainly caused by alteration of cell elongation. Expression analyses showed that *OsAGO17* was highly expressed in young panicles and nodes, which was consistent with the expression pattern of OsmiR397b. SRNA sequencing, stem‐loop RT‐PCR and sRNA blotting showed that the expression of OsmiR397b was reduced in *ago17* and enhanced in the *OsAGO17* OE lines. Four OsmiR397b target *laccase* (*LAC*) genes showed complementary expression patterns with *OsAGO17* and OsmiR397b. Combined with the results of immunoprecipitation (IP) analysis, we suggested that OsAGO17 formed an RISC with OsmiR397b and affected rice development by suppression of *LAC* expression. In conclusion, OsAGO17 might be a critical protein in the sRNA pathway and positively regulates grain size and weight in rice.

## Introduction

Argonaute (AGO) proteins are widely distributed in eukaryotes and are the primary components of the RNA‐induced silencing complex (RISC) (Hutvagner and Simard, 2008). AGOs can function in both transcriptional and post‐transcriptional gene silencing (Song *et al*., 2004) and have three main domains: PIWI, MID (middle) and PAZ. The catalytic PIWI domain has an RNase‐H‐like fold, with three key metal‐coordinating residues, Asp‐Asp‐Glu (DDH) (Hutvagner and Simard, 2008). The DDH motif potentially acts as a conserved catalytic centre of silencing effector complexes (Yigit *et al*., 2006). AGOs repress target gene expression by various mechanisms. In animals, AGOs cause RNA destabilization or sequestration and prevent targeted RNAs from producing proteins (Bartel, 2009). In ciliates, AGOs could directly facilitate developmentally programmed DNA elimination (Lee and Collins, 2006). AGOs also directly catalyse targeted RNA cleavage in all eukaryotes (Bartel, 2009; Song *et al*., 2004). In plants, AGO‐induced RNA cleavage can be followed by either DNA methylation or heterochromatin restructuring (Law and Jacobsen, 2010).

Based on functional domains, eukaryotic AGO proteins are divided into two major subfamilies: AGO and PIWI (Vaucheret, 2008). AGOs are highly multiplicate in animals and insects, in which both kinds of AGO proteins are present (Vaucheret, 2008). In plants, all AGOs belong to the AGO subfamily (Hutvagner and Simard, 2008; Vaucheret, 2008). Almost all AGO proteins in flowering plants can be classified into three clades based on phylogenetic analyses (Zhang *et al*., 2015), such as in *Arabidopsis*: clade I AGO1/5/10; clade II AGO2/3/7; and clade III AGO4/6/8/9 (Vaucheret, 2008). The AGO family has expanded during evolution. In multicellular green algae, less than three AGOs have been found, while in mosses and lycophytes, the number has increased to seven (Zhang *et al*., 2015). The family is even larger in flowering plants, with 10 AGOs in *Arabidopsis*, 14 AGOs in *Sorghum*, 17 AGOs in maize, 19 AGOs in rice and 21 AGOs in soybean (Kapoor *et al*., 2008; Liu *et al*., 2014; Qian *et al*., 2011). Numerous gene duplications may be the main reason for the expansion of this family. For example, 2 AGO4 homologues, 4 AGO1 homologues and 5 AGO5 homologues are found in rice (Kapoor *et al*., 2008). The high number of homologues may induce functional diversification of AGOs (Singh *et al*., 2015; Zhang *et al*., 2015).

AGOs regulate plant growth and development with various small RNAs (sRNAs). AtAGO1 regulates leaf development and cooperates with AtAGO10 to regulate floral stem cell termination through miR172 and miR165/166 (Ji *et al*., 2011). AtAGO10 competes with AtAGO1 to bind miR166/165 to repress the expression of HD‐ZIP III genes, thus maintaining the shoot apical meristem (Zhu *et al*., 2011). In response to bacterial infection, AtAGO2 binds miR393b, which targets a Golgi‐localized SNARE gene, *MEMB12* (Zhang *et al*., 2011). *AtAGO5* is specifically expressed around megaspore mother cells and binds siRNA from somatic cells to inhibit pathways required for initiation of the mitosis of megagametophytes (Tucker *et al*., 2012). *AtAGO7* plays an important role in adult‐phase vegetative traits (Hunter *et al*., 2003). *AtAGO9* is expressed in reproductive companion cells; this protein restricts the differentiation of gametophyte precursors and ensures the proper differentiation of female gametes (Olmedo‐Monfil *et al*., 2010). Down‐regulation of *OsAGO4a* and *OsAGO4b* causes dwarfism, shortened panicle length and decreased branching by regulating gibberellin (GA) and brassinosteroid (BR) homoeostasis‐related genes (Wei *et al*., 2014). *OsMEL1*, a gene homologue of *AtAGO5*, was specifically detected in germ cells and regulates the meiotic chromosome modification of these cells (Komiya *et al*., 2014; Nonomura *et al*., 2007). An *AtAGO10* homologue, *OsPNH1*, also regulates SAM and leaf development (Nishimura *et al*., 2002). Overexpression of *OsAGO7* induces upward curling of leaves (Shi *et al*., 2007). *OsAGO18* plays an important role in antiviral defence pathways through binding miR168 to regulate *OsAGO1* or binding miR528 to cleave L‐ascorbate oxidase mRNA (Wu *et al*., 2015, 2017).

Ideal plant height and grain size are two important components of grain yield in rice (Vanhaeren *et al*., 2016). Recently, some miRNAs have been reported to influence rice yield. OsmiR156 cooperates with the target gene *OsSPL14* to define the ideal plant architecture and influence the grain yield, and there exist complex gene networks that are regulated by miR156/SPL, miR172/AP2 and miR529 (Jiao *et al*., 2010; Wang *et al*., 2012, 2015). OsmiR397b is highly expressed in young panicles, increasing panicle length, plant height and grain size by down‐regulating the expression of the target gene *OsLAC*. Meanwhile, *OsLAC* can affect the sensitivity of BR (Zhang *et al*., 2013). OsmiR398 promotes rice yield by increasing the grain number per panicle, grain size and grain weight (Zhang *et al*., 2017a,2017b). OsmiR408 increases panicle branching and grain number to promote rice yield and targets the uclacyanin gene *OsUCL18* (Zhang *et al*., 2017a,2017b). AGOs are essential for miRNA‐mediated regulation of target gene expression, but it remains unclear which AGO affects these yield‐related characteristics via accumulation of sRNA.

OsAGO17 belongs to clade one in rice and has no paralog in rice or other species (Kapoor *et al*., 2008; Zhang *et al*., 2015). In our study, we investigated the expression pattern of *OsAGO17* by multiple methods, and the results showed that OsAGO17 was a ubiquitously expressed gene, with the highest expression levels observed in stems, young panicles and young seeds. To understand the function and mechanism of OsAGO17, *OsAGO17* overexpressing transgenic plants (OE lines) and two types of down‐regulating mutants, namely, RNAi transgenic plants (RNAi lines) and *ago17* mutants, were constructed using the CRISPR‐Cas9 system. Grain size and 1000‐grain weight were clearly increased in the OE lines compared with the wild type (WT) and ZH11 (*Oryza sativa* ssp. *japonica* cv. Zhonghua 11). Historical analysis indicated that this gene influenced spikelet size by cell elongation. Further analysis showed that OsAGO17 may suppress the expression of *LAC* via accumulation of OsmiR397b, which regulates seed and stem development in rice. These results imply that OsAGO17, a putative AGO protein, may play an important role by affecting spikelet size and may thereby affect the grain size and weight of rice. The discovery of OsAGO17 may facilitate the regulation of seed size and weight, and this gene can be effectively used in crop breeding programmes.

## Results

### Expression pattern and subcellular localization of *OsAGO17*

**Figure 1:**
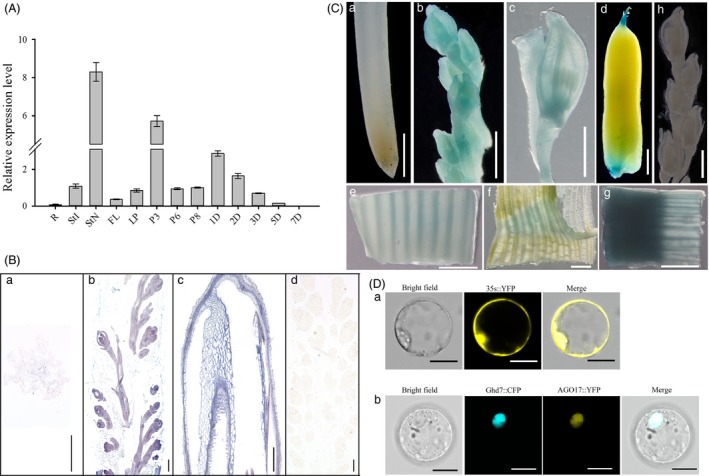
Spatial and temporal expression pattern of *OsAGO17*. (A) QRT‐PCR analysis of *OsAGO17*. Root (R), internode (StI), node (StN), flag leaf (FL) and leaf pulvinus (LP) at jointing stage; P3, P6 and P8, panicles at stage 3, 6 and 8, respectively; 1D‐7D, seeds at 1, 2, 3, 5 and 7 DAP. Data are the mean ± SE (*n* = 3). (B) RNA *in situ* hybridization of OsAGO17. a. Root. b. Panicle at stage 3. c. Developing glume. d. Negative control. Bar = 100 μm. (C) GUS staining. a. Root; b. panicles at stage 4; c. spikelet at p6 stage; d. 3 DAP seed; internode (e), leaf pulvinus (f) and node (g) at jointing stage; h. negative control. Bar = 1 cm. (D) Subcellular location of OsAGO17. a. YFP protein signal promoted by 35S. b. YFP represents OsAGO17 and CFP represents OsGhd7. Bar = 15 μm.

QRT‐PCR was performed to investigate the expression pattern of *OsAGO17* in different tissues of ZH11, and *OsAGO17* was found to be ubiquitously expressed. Among vegetative organs, *OsAGO17* was expressed at significantly higher levels in flag leave and stems than in roots. Meanwhile, high levels of mRNA transcripts were accumulated in early developing panicles and seeds at 2–3 days after pollination (DAP) (Figure 1A). *In situ* hybridization was performed to detect the expression of *OsAGO17* in the panicles. The results showed that *OsAGO17* was highly expressed in young panicles and developing glumes (Figure 1B).

To gain insight into the spatiotemporal expression pattern of *OsAGO17*, promoter analysis was performed in transient assays using a glucuronidase (GUS) reporter gene. The results of GUS staining analysis of 6 independent lines matched well with the quantitative real‐time PCR (qRT‐PCR) results (Figure 1C). The signal was strong in young panicles and lemmas of spikelets. Further analysis revealed that the expression level of *OsAGO17* in leaves and stems was much higher than that in roots, particularly in leaf pulvinae and vascular bundles of stems, and exceedingly high in stem nodes. In addition, *OsAGO17* was expressed at the ends of the micropyles or chalazas in 3 DAP seeds (Figure 1C).

For subcellular localization analysis, the protoplast transient gene expression vector pM999 with the CaMV 35S promoter was used in this study. OsAGO17 was fused with yellow fluorescent protein (YFP), while OsGhd7 was fused with cyan fluorescent protein (CFP) as a nuclear localization signal (Xue *et al*., 2008). The YFP signal of the OsAGO17 protein colocalized with the CFP signal of the OsGhd7 protein in the nucleus, while the signal of 35s::YFP was detected throughout the cell. This observation revealed the nuclear localization of OsAGO17 (Figure 1D).

### Production of *OsAGO17*‐overexpressing and *OsAGO17*‐suppressing lines

**Figure 2:**
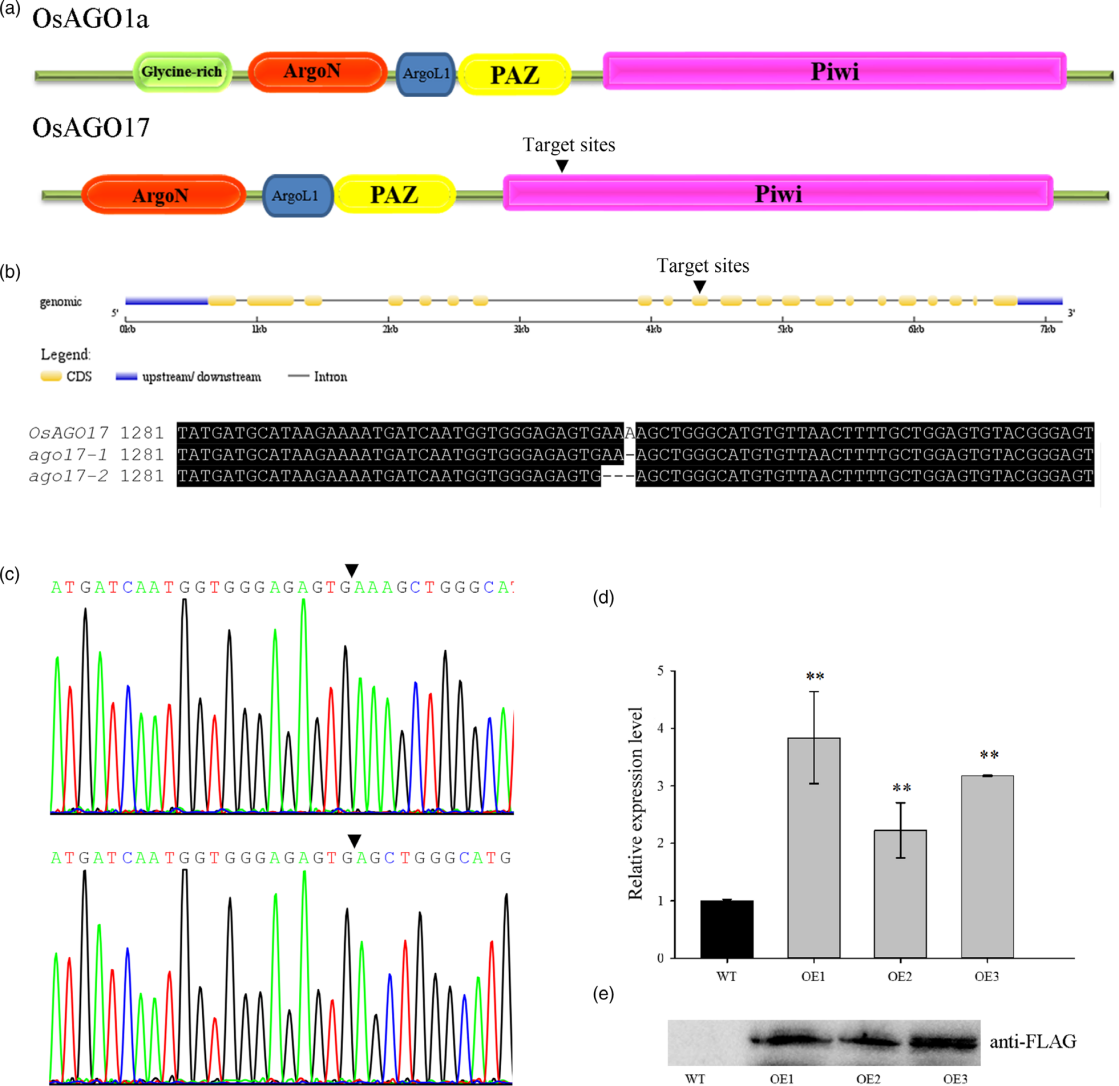
Characterization of *OsAGO17* mutants and transgenic overexpression plants. (a) Structural schematic diagram of OsAGO1a and OsAGO17. The inverted triangle shows the positions of the target region. (b–c) Sequencing of *ago17‐1* and *ago17‐2* showing the target region. (d) Relative expression levels in young panicles of three OE lines. (e) Western blot of young panicles from three OE lines using anti‐FLAG. Data are the mean ± SE for three replicates. **P < 0.01 by Student's *t*‐test.

*OsAGO17* encodes a putative 100‐kDa protein with a PAZ domain, a PIWI domain and an Argonaute‐specific N‐terminal domain, but the glycine‐rich region found at the N‐termini of OsAGO1s and OsMEL1 was absent in OsAGO17 (Figure 2A, Figure S1A). The conserved catalytic residues DDH in the PIWI domain were replaced by HDR in OsAGO17 (Figure S1B). Therefore, this change might affect the function of OsAGO17.

To study the function of OsAGO17, the CRISPR/Cas9 genome editing system was used to obtain specific mutations. A sequence (ATGGTGGGAGAGTGAAAAGC) from the tenth exon was chosen as the single‐guide RNA (sgRNA) target site. Twenty transgenic seedlings were obtained, and five *T*_0_ (C5, C9, C13, C17, and C19) heterozygous plants were identified by decoding the sequencing chromatograms. Two kinds of knockout homozygous mutants were obtained in *T*_1_ plants, namely, a one‐adenine deletion mutant and a three‐adenine deletion mutant in the tenth exon (Figure 2B–C). Two independent lines (C17‐3 and C19‐2) from T_0_ plants (C17 and C19) (Table S1) were grown in the field for investigation and renamed *ago17‐1* and *ago17‐2* (Figure 2B), respectively.

We also obtained *OsAGO17* RNAi lines. Twelve T_0_ plants exhibited low expression of *OsAGO17*, and three plants (R3, R8, and R15) were selected for the next generation. Finally, three independent RNAi lines (R3‐13, R8‐22 and R15‐7) with low expression levels were grown for functional analysis and renamed R1, R2 and R3 (Table S2). The expression of 4 *OsAGO1s* was investigated, and no significant changes were observed in the RNAi lines (Figure S2A–B).

We also generated OE lines of *OsAGO17*. Twenty T_0_ plants were obtained, and 10 successful transformants were confirmed by PCR. Three independent OE lines (OE11‐23, OE13‐13 and OE19‐2) from T_0_ plants (OE11, OE13 and OE19) with high expression levels were advanced to the T_2_ generation for subsequent research and renamed OE1, OE2 and OE3, respectively (Figure 2D, Table S3). Western blot results confirmed the translation of OsAGO17 in the OE lines (Figure 2E). All transgenic WT plants showed no obvious change compared with ZH11 (Tables S1–S2); thus, we used pure ZH11 as the WT.

### *OsAGO17* might positively regulate grain size and stem development in rice

**Figure 3:**
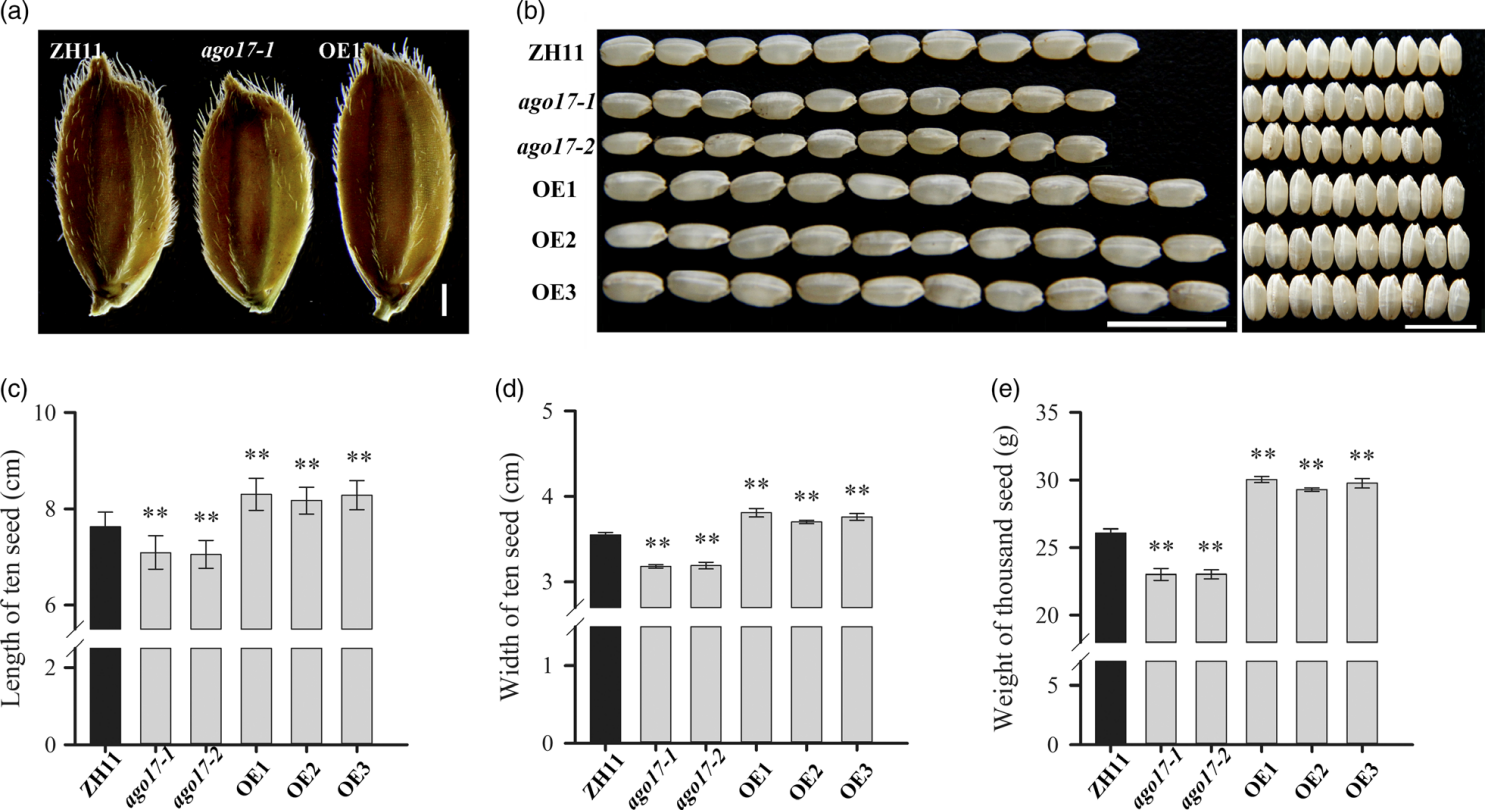
Statistical analyses of grain size and weight in ZH11, *ago17* and OE lines of *OsAGO17*. A–B. Grain (a) and dehulled grains (b) of ZH11, *ago17‐1* and OE1. Bar = 1 mm (a). Bar = 1 cm (b). c–e. Length (c) and width (d) of ten seeds and weight of 1000 seeds (e) of the ZH11, *ago17* and OE lines. The data are presented as the mean ± SE, *n* = 30, **P < 0.01, by Student's *t*‐test.

We investigated the influence of *OsAGO17* on yield‐related traits and first found that the grain size showed obvious differences among the ZH11, *ago17* and OE lines. Compared with ZH11, the grain length (–7.2%) and grain width (–10.4%) substantially decreased in *ago17*, while in the OE lines, the seeds showed a significant increase in grain length (+8.3%) and grain width (+6.8%) (Figure 3A–D). In addition, the 1000‐grain weight increased substantially in the OE lines (+13.9%), while a prominent decrease was detected in *ago17* (–12.3%) (Figure 3E). Other yield‐related traits were also examined (Table S4). The number of primary branches and tillers did not change among the lines. In *ago17*, the panicle length (–13.0%), spikelet number per panicle (–30.9%), setting rate (–28.8%) and grain yield per plant (–45.3%) were obviously reduced compared with those in ZH11. In contrast, in the OE lines, the spikelet number per panicle (–15.8%) and setting rate (–13.7%) also decreased, and the reduction was weaker than that in *ago17*. However, the panicle length showed a small increase (+5.4%), and the grain yield per plant was similar to that in ZH11. Additionally, the seeds of the *ago17* and OE lines showed obvious chalkiness (Figure S3A–B).

**Figure 4:**
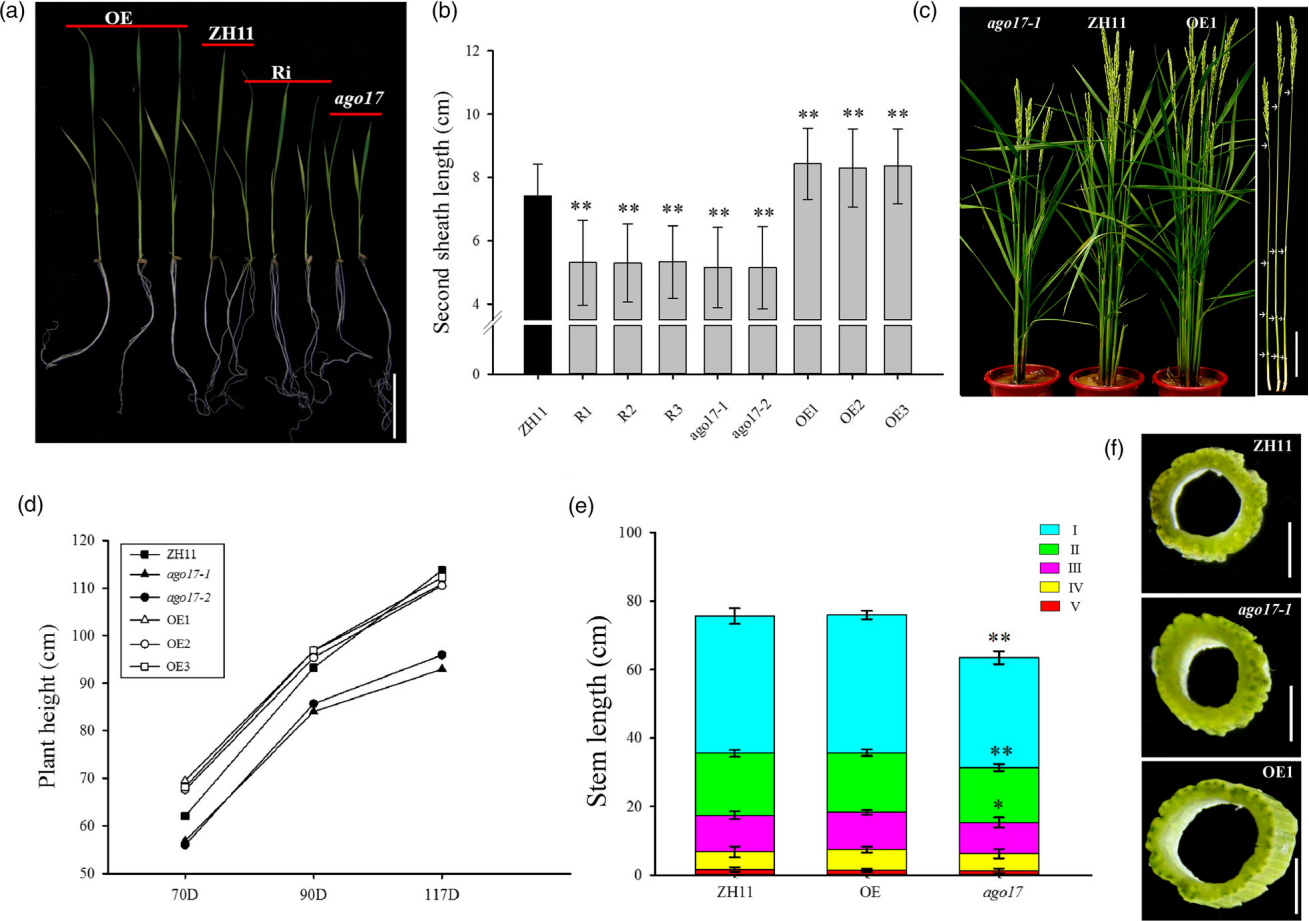
Plant height and stem development alteration in *ago17* and the *OsAGO17* OE lines. (a–b) Phenotype observation of three‐week‐old seedlings (a) and second sheaths (b). Bar = 5 mm. (c) Phenotype observation of whole mature plants (left) and stems (right). Bat = 10 cm. (d) Plant height at 70, 90 and 117 Days. (e) Different internode lengths in the ZH11, *ago17* and OE lines. I, II, III, IV and V indicate the first, second, third, fourth and fifth internodes, respectively. (f) Comparison of the first internode cross section. Bar = 1 mm. Data are presented as the mean ± SE, *n* = 30, **P < 0.01, *P < 0.05, by Student's *t*‐test.

Morphological differences were also observed between ZH11 and the transgenic lines at the vegetative stage. At the seedling stage, *ago17* exhibited obvious dwarfism (–30%) compared with ZH11, while in the OE lines, the plants were very strong and increased almost 15% in height (Figure 4A–B). Throughout the growth season, *ago17* remained shorter than ZH11. In the OE lines, the plant heights were much greater than that of ZH11 until the heading stage (Figure 4C–D). The internode length was evaluated, and only the first three internodes were shortened in *ago17* (Figure 4C, E). The cross section of the internode showed that the stems of the OE lines were much thicker and sturdier than that of ZH11, and the stem of *ago17* was slightly smaller than that of ZH11 (Figure 4F).

The phenotypes of the RNAi lines were similar to those of *ago17*, such as dwarfism, shortened stems and panicles, small spikelets and seeds, and low setting rates. The phenotypes of the RNAi lines were consistent with the expression of *OsAGO17* (Figure S2). In addition, the developing hulled seeds were consistently small in the RNAi lines and *ago17* (Figure S4). The total protein and starch levels in the seeds of the RNAi lines were similar to those in the WT (Figure S3C–D). All these results showed that *OsAGO17* could positively regulate grain size and weight in rice.

### *OsAGO17* promoted cell elongation to regulate organ development in rice

**Figure 5:**
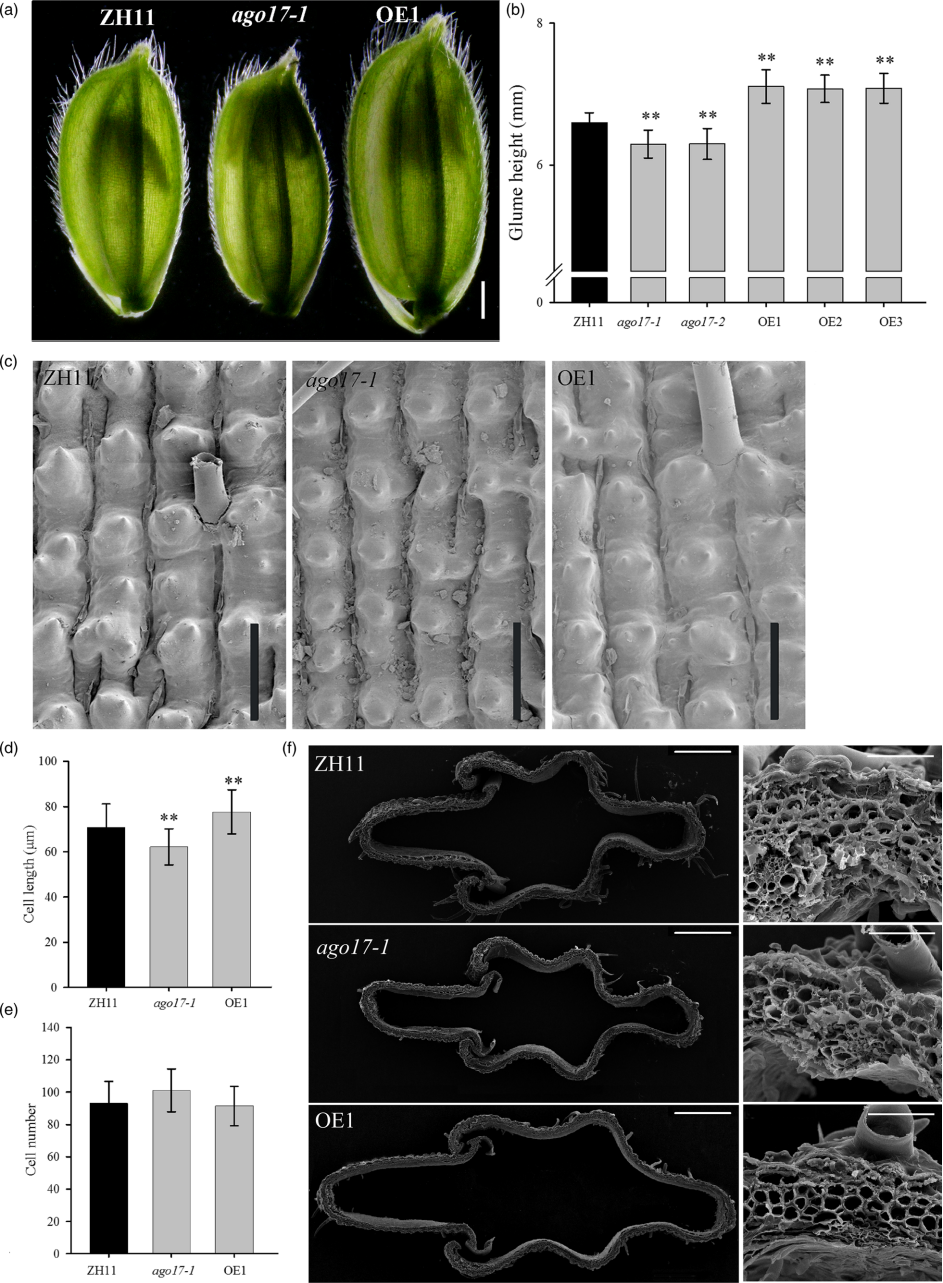
OsAGO17 affects cell elongation in rice spikelets. (a) Spikelet at a day before heading of the ZH11, *ago17‐1* and OE lines, bar = 1 mm. (b) Glume length, *n* > 100 flowers from 5 panicles. (c) Epidermal layers from middle sections of glumes in mature seeds, bar = 100 μm. (d–e) Cell length (d) and cell number (e) of epidermal layers, *n* = 15. f. Cross section of the middle parts of glumes one day before flowering, as observed by SEM. Left bar = 500 μm. Right bar = 50 μm. The data are presented as the mean ± SE, **P < 0.01, by Student's *t*‐test.

Considering the effect on seed size from glumes, we examined the spikelets 1 day before heading. The spikelet of *ago17* was shorter and narrower than that of ZH11, but those of the OE lines were much longer and wider than that of ZH11 (Figure 5A). The measurement data indicated that the length of spikelet decreased approximately 8% in *ago17* and increased 8% in the OE lines compared with that of ZH11 (Figure 5B). The cells in the outer epidermis of mature grains were investigated by SEM. The cells of *ago17* were smaller than those of ZH11, while the cells of the OE lines were longer and wider than those of ZH11 (Figure 5C). Based on statistics, compared with the cell length in ZH11, the cell length along the longitudinal axis decreased 10% in *ago17* but increased 7% in the OE lines (Figure 5D). The cell number (spikelet length divided by the cell length) showed no significant changes among the lines (Figure 5E). Cross sections of the central parts of the spikelets showed that the spikelets of the OE lines possessed larger areas and contained substantially larger cells than those of ZH11, while the spikelets of *ago17* were much smaller than those of ZH11 (Figure 5F).

**Figure 6:**
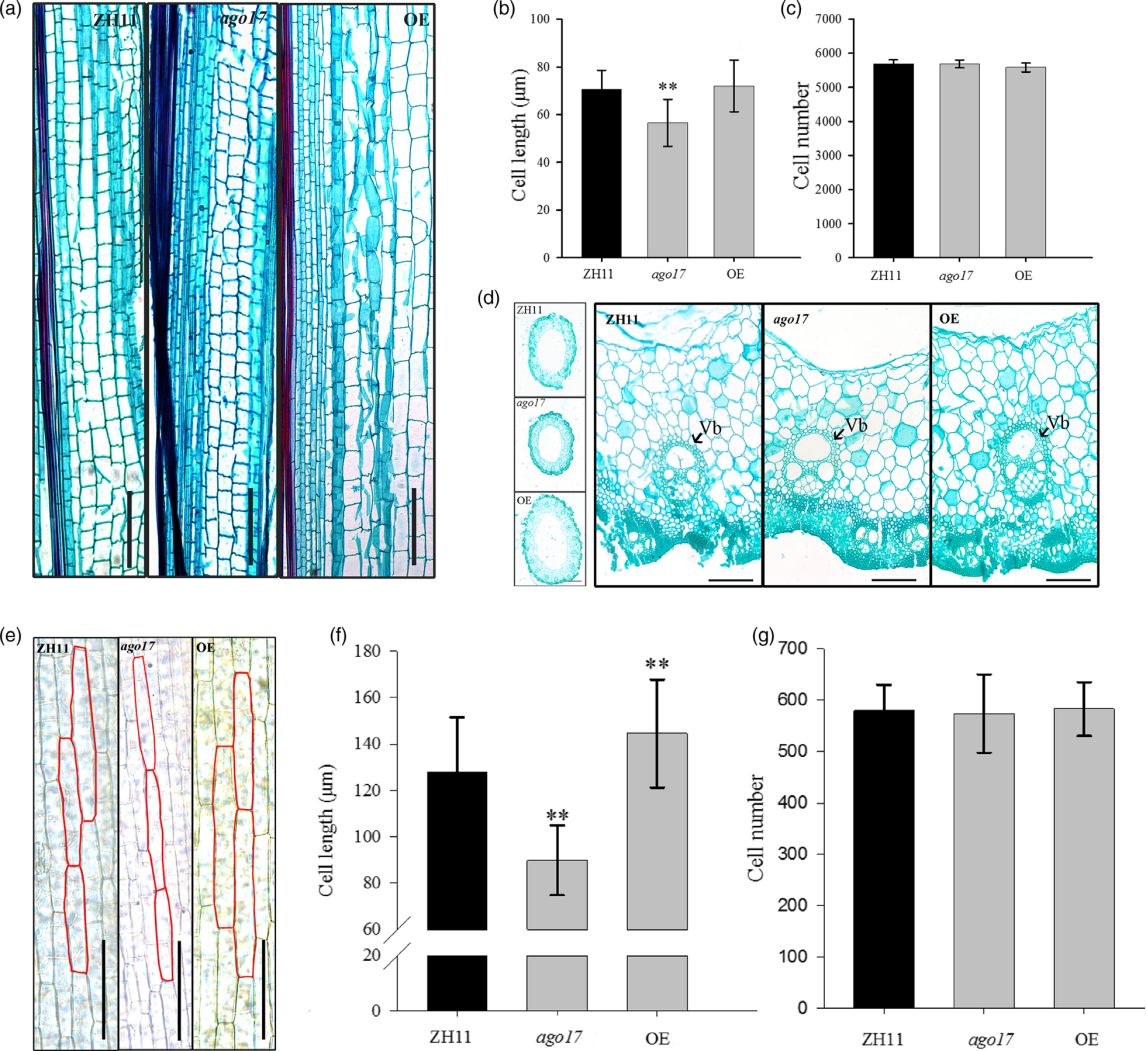
OsAGO17 affects cell elongation in the stem and sheath. (a) Longitudinal section of the first internode of a mature stem, bar = 200 μm. (b–c) Cell length (b) and cell number (c) of stem cortex cells. (d) Comparison of first internode cross sections, left bar = 5 mm and right bar = 100 μm. Vb. Vascular bundle. (e) Histological observation of the second sheath of the ZH11, *ago17* and OE lines, bar = 100 μm. (f–g) Cell length (f) and cell number (g) of the second sheath. The data are presented as the mean ± SE, *n* = 15 sections, **P < 0.01, by Student's *t*‐test.

Based on histological observation of stems, we found that along the longitudinal axis, the cells were much shorter in *ago17* than in ZH11, while the cells in the OE lines were much wider than those in ZH11 but with similar lengths (Figure 6A). Based on statistical analysis, the cell length was significantly reduced (–19.5%) in *ago17* (Figure 6B), and the cell number showed no significant change (Figure 6C). Moreover, the stems of the OE lines were obviously thicker and sturdier than those of the other lines, and the vascular bundles were more developed along the cross section than those of the other lines (Figure 6D). We also found that in *ago17,* the cell length of the second leaf sheath decreased compared with that of ZH11, and this value increased in the OE lines, which was consistent with the plant height at the seedling period (Figure 6E–F). The cell lengths in stems and glumes were lower in the *OsAGO17* RNAi lines than in the WT (Figure S5). Therefore, we considered that *OsAGO17* might affect organ size by regulating cell elongation in rice.

Additionally, we tested the expression of a few genes that control grain size in rice (Figure S6). The expression of the cell proliferation‐related genes *GS5*,* GSN1*,* GW2* and *SRS3* showed no significant change among the three samples. The expressions levels of the cell expansion‐related genes *GW7*,* PGL1* and *PGL2* were comparable among *ago17*, the OE lines and ZH11. Considering the influence of GA and BR on cell expansion, the expression levels of GA‐ and BR‐related genes were tested, and differential expression of these genes was observed among *ago17*, the OE lines and ZH11. Therefore, we speculated that *OsAGO17* might participate in the plant hormone pathway to regulate cell expansion.

### OsAGO17 affected the expression of miRNA

**Figure 7:**
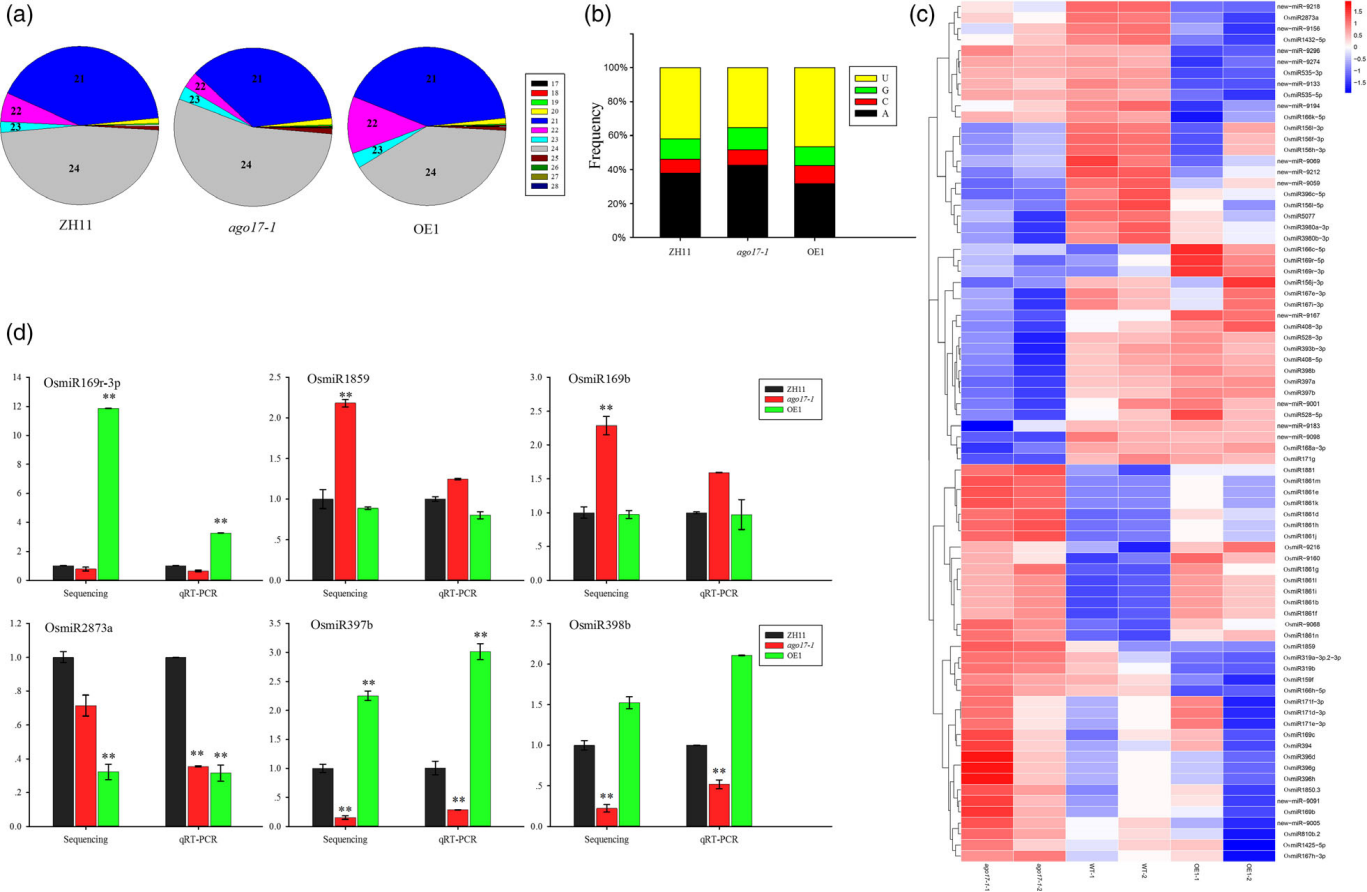
Effects of the changes in *OsAGO17* expression on miRNA abundance in rice. (a) sRNA length distribution. Blue, 21 nt sRNA; purple, 22 nt sRNA; and grey, 24 nt sRNA. (b) Distribution of the 5′‐terminal nucleotide of sRNA. Black, adenine; red, cytosine; green, guanine; and yellow, uridine. (c) Expression comparison of miRNAs between *ago17‐1* and ZH11 or OE1 and ZH11. (d) Stem‐loop RT‐PCR of miRNA accumulation. The data are presented as the mean ± SE, *n* = 3, **P < 0.01, by Student's *t*‐test.

Considering the important role of AGO proteins in the sRNA pathway, sRNA sequencing was performed in *ago17‐1*, OE1 and ZH11. sRNAs that were 17‐28 nt in length were mapped to the rice genome and kept for analysis after removing tRNA, rRNA, snRNA and snoRNA sequences. Based on the length distribution, the 21‐24 nt sRNAs were more abundant than the others, and the levels of 21 nt and 22 nt sRNAs were 5.4% and 2.6% lower in *ago17‐1* than in ZH11, respectively, but 0.5% and 5.8% higher in OE1 than in ZH11, respectively (Figure 7A). We also analysed the 5′‐termini of these sRNAs, and most of the sRNAs contained either adenine or uridine at the 5′‐terminus. The percentage of 5′‐terminal uridine was 6.7% lower in *ago17‐1* than in ZH11 and 4.7% higher in OE1 than in ZH11 (Figure 7B).

To check the variation in miRNA levels, we mapped the reads to the miRbase database. First, we analysed the differential expression of known OsmiRNAs. Among the 713 OsmiRNAs in miRbase, 592 OsmiRNAs were detected in our experiment. Compared with the levels in ZH11, in *ago17‐1*, 12 were down‐regulated and 18 were up‐regulated, and in OE113, 7 were down‐regulated. Additionally, 310 potential novel OsmiRNAs were predicted in our data pool, and a few exhibited altered levels in *ago17‐1* (6 down‐regulated, 4 up‐regulated) and in OE1 (1 up‐regulated, 6 down‐regulated) (Figure 7C, Tables S5–S6). To verify the change in expression observed in the sRNA sequencing data, stem‐loop RT‐PCR was performed on six miRNAs, and the results were consistent with those of sRNA sequencing (Figure 7D).

In addition, there were 457 ta‐siRNAs in the database, but only 60 ta‐siRNAs were found in our sRNA libraries. These ta‐siRNAs had very low counts and showed no significant changes among the three samples. Based on PlantNATsDB, we mapped our sequences to the 409790 NAT pairs to predict nat‐siRNAs. Ninety‐nine per cent of the NAT pairs exhibited a nat‐siRNA match, but the expression levels of more than 99.6% of the nat‐siRNAs appeared to be unchanged. Therefore, we inferred that OsAGO17 might influence only the miRNA pathway and had no function in the ta‐siRNA or nat‐siRNA pathway.

### *OsAGO17* regulates grain size and plant height with miR397b

MiRNAs that were simultaneously down‐regulated in *ago17‐1* and up‐regulated in OE1 were used for analyses of pathways related to *OsAGO17*. It was found that the level of OsmiR397b decreased in *ago17‐1* (fold change (FC) = 0.15, adjusted *P*‐value (padj) < 0.01) and increased in OE1 (FC = 2.25, *P*adj < 0.01) (Table S5–S6). A previous report (Zhang *et al*., 2013) suggested that OsmiR397b overexpressing plants showed strong seedlings and large seeds, similar to *OsAGO17* overexpressing plants; while overexpression of *OsLAC*, a target gene of OsmiR397b, showed small grains and semi‐dwarf plants, similar to *ago17* and the *OsAGO17* RNAi lines (Figures 3 and 4). Therefore, OsmiR397 may play an important role in the *OsAGO17*‐mediated pathway in rice development.

**Figure 8:**
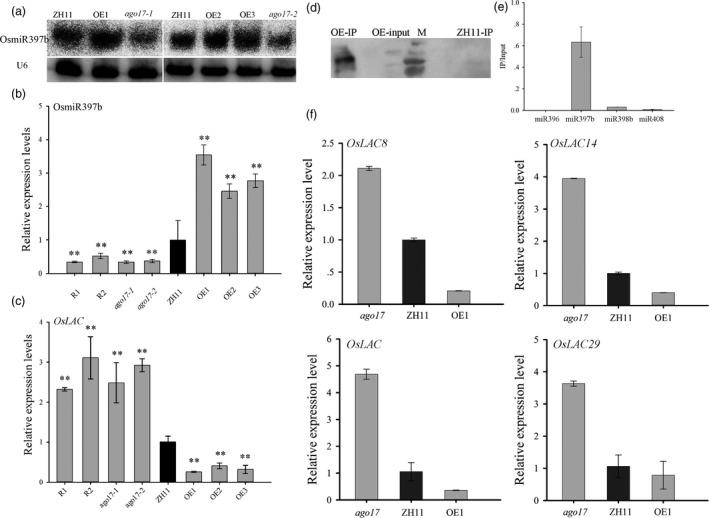
OsAGO17‐mediated down‐regulation of miR397b and change in expression of its target genes. (a) Northern blotting of OsmiR397b in three‐week‐old seedlings (left) and young panicles (right). Up, OsmiR397b. Down, U6. (b–c) Relative expression levels of OsmiR397b (b) and its target gene *OsLAC* (c) in ZH11 and transgenic plants in young panicles. The data are presented as the mean ± SE, *n* = 3, **P < 0.01, by Student's *t*‐test. (d–e) IP analysis showed that OsAGO17 may accumulate OsmiR397. Western blot of the IP precipitate with anti‐FLAG (d) and stem‐loop RT‐PCR (e). Y axis, IP/input. (f) Expression of other OsmiR397b downstream target genes.

To determine whether OsmiR397b participates in OsAGO17 function, stem‐loop RT‐PCR and sRNA blotting were performed. The results showed that the accumulation of OsmiR397b was reduced in *ago17* and increased in all of the OE lines (Figure 8A–B). In addition, in *ago17,* the expression of *OsLAC* was significantly higher than that in ZH11, while in the OE lines, the expression level of *OsLAC* was greatly decreased (Figure 8C).

Immunoprecipitation (IP) analysis with anti‐Flag in the OE lines was performed to test whether OsAGO17 associates with OsmiR397b. First, Western blotting showed that the OsAGO17 proteins precipitated well (Figure 8D). Then, RNA was isolated from the precipitate for stem‐loop RT‐PCR. The results indicated that OsmiR397 was conspicuously enriched compared with OsmiR398 or OsmiR408, while OsmiR396 showed no enrichment. Thus, we hypothesized that OsAGO17 may accumulate OsmiR397b to regulate rice growth and development. Additionally, the expression levels of other OsmiR397b‐targeted LAC genes were tested. Three target genes, namely, *OsLAC8*,* OsLAC14* and *OsLAC29*, showed similar changes as *OsLAC* (Figure 8E. Table S7), and according to the CREP database, the expression levels of *OsLAC8*,* OsLAC* and *OsLAC29* were inversely correlated with that of *OsAGO17* (Figure S7). Therefore, OsAGO17 and OsmiR397b may suppress the expression of other target genes to facilitate rice development.

## Discussion

In our study, we identified the function of the putative AGO protein OsAGO17 from rice. When *OsAGO17* was overexpressed, the grain size and grain weight significantly increased, and the grain quality changed. In down‐regulated mutants, the grain size and grain yield were greatly reduced. Thus, our studies may help with molecular breeding of rice in the future with a focus on improving grain yield and seed quality.

Many factors influence grain weight, such as grain size and degree of grain filling (Li and Li, 2016; Zhou *et al*., 2017). *GS5* (Li *et al*., 2011), *GW2* (Song *et al*., 2007), and *GSN1* (Guo *et al*., 2018) have been reported to control grain weight by regulating the cell proliferation of spikelets in rice. In our research, the changes in expression showed were not comparable among ZH11, the OE lines and *ago17*. *PGL1* (Heang and Sassa, 2012) and *PGL2* (Heang and Sassa, 2012) control grain weight by regulating cell expansion and spikelet size in rice, and there were some differences in the expression of these genes. Furthermore, obvious changes in cell size were found among ZH11, the OE lines and *ago17*. These results suggest that OsAGO17 affects rice grain weight by influencing spikelet cell size. Photosynthate production in stems, translocation through the vascular bundle and accumulation in grains are prerequisites for increased grain filling rates (Khush, 1995). *OsAGO17* OE lines exhibit increased plant heights during the vegetative growing stage, with thick and sturdy stems and developmental vascular bundles, which facilitate energy accumulation in plants; these features are important because they guarantee large seeds. The increased glumes provided enough space for endosperm growth.

MiRNAs have been identified as important regulators of grain size and yield in rice; among these miRNAs, miR397, miR398 and miR408 positively regulate grain size (Zhang *et al*., 2013, 2017a,b). AGO is an essential protein that is involved in gene expression regulation through miRNAs (Bartel, 2009), and it is highly likely that at least one AGO protein is involved in grain size regulation in rice. In our transgenic plants, the change in expression of OsmiR397b was similar to that of *OsAGO17*. Additionally, OsmiR397b was highly expressed in young panicles, spikelets and stem nodes (Zhang *et al*., 2013), and in our study, *OsAGO17* showed a similar expression pattern. *OsAGO17* increases grain size and OsmiR397b levels (Zhang *et al*., 2013). The IP analysis suggested that OsAGO17 could affect rice development with OsmiR397b by suppressing target gene expression.

In *Arabidopsis*, miR397 targets *LAC2*,* LAC4* and *LAC17* and responds to low copper availability (Abdel‐Ghany and Pilon, 2008). *LAC* genes may cooperate to regulate plant development, that is the *LAC4* and *LAC17* genes are involved in the lignification of *Arabidopsis* stems (Berthet *et al*., 2011). In our study, three *LAC* genes showed the same change in expression as *OsLAC*, and the expression patterns of these genes were complementary to that of *OsAGO17*. *OsLAC14* is a homologue of *OsLAC* (Liu *et al*., 2017). Thus, *OsLAC* may not be the only target gene of OsmiR397b in the regulation of grain size, and *OsAGO17* may affect grain size by regulating the expression of other LAC genes.

The expression levels of some miRNAs were changed in the *OsAGO17* transgenic plants, such as OsmiR156j, OsmiR396c, OsmiR398b and OsmiR408, all of which have been reported to regulate seed development in rice (Jiao *et al*., 2010; Zhang *et al*., 2017a,b), so there may be a complex regulatory network associated with OsAGO17. In addition, OsmiR397, OsmiR398 and OsmiR408 also play an important role in abiotic and biotic stress responses (Jia *et al*., 2009; Lu *et al*., 2011; Pan *et al*., 2017). *OsLAC8* and *OsLAC29* were significantly induced by salt stress in both salt‐sensitive cultivars (Liu *et al*., 2017). Therefore, we hypothesized that *OsAGO17* may respond to stress in rice.

In plants, the length and 5′‐terminal nucleotide of an sRNA influence the association of the sRNA with the AGO protein (Hutvagner and Simard, 2008). AtAGO1 associates with 21 nt miRNA with a 5′‐terminal uridine, and three OsAGO1 proteins (OsAGO1a, OsAGO1b and OsAGO1c) associate with 21 nt miRNA with a 5′‐terminal uridine (Wu *et al*., 2009). AtAGO5 specifically binds to 21 nt siRNA with a 5′‐terminal cytosine, and the AtAGO5 homologue MEL1 in rice binds to 21 nt phasiRNAs with 5′‐terminal cytosines (Komiya *et al*., 2014). AtAGO7 and its homologue ZmAGO7 all accumulate miR390 to regulate TAS3‐derived ta‐siRNA biogenesis (Montgomery *et al*., 2008). AtAGO4, AtAGO6 and AtAGO9 in the same clade all prefer to sort 24 nt siRNA with a 5′‐terminal adenosine (McCue *et al*., 2015; Zhang *et al*., 2015). In summary, the sRNA that associates with the AGO protein is relatively well conserved.

As a typical AGO protein in the AGO1 clade based on phylogenetic analysis, *OsAGO17* might also participate in the sRNA pathway. According to the results of sRNA sequencing analysis, sRNAs with a 5′‐terminal uridine were depleted in *ago17‐1* but enriched in OE1. In Chlamydomonas, 98% of CrAGO3 associates with a sRNA with a 5′‐terminal uridine, and upon loss of the CrAGO3 protein, the abundance of the sRNA with a 5′‐terminal uridine decreased (Voshall *et al*., 2015; Yamasaki *et al*., 2016). Therefore, we presumed that OsAGO17 may prefer to associate with sRNAs with a 5′‐terminal uridine, such as AGO1. Considering the length distribution, the level of 21 nt sRNA was decreased in *ago17‐1* and slightly increased in OE1, while the level of 22 nt sRNA was increased in OE1 and slightly decreased in *ago17‐1*, so we inferred that OsAGO17 may sort 21 or 22 nt sRNA. Based on IP analysis, OsAGO17 may accumulate miR397b, and the most highly accumulated sequence of miR397 was 22 nt with a 5′‐terminal uridine. Thus, OsAGO17 may accumulate 22 nt sRNA with a 5′‐terminal uridine.

## Methods

### Plant material and growth conditions

The RNAi constructs with 376‐bp‐specific nucleotides from the *OsAGO17* coding sequence were amplified from young‐panicle‐derived cDNA of ZH11. Then, the sequences were introduced into the target vector pDS1301 as previously reported (Chu *et al*., 2006).

The specific sgRNA targeting the *OsAGO17* gene was designed using the web‐based tool CRISPR‐P (http://crispr.hzau.edu.cn/CRISPR/) and introduced into U3 by overlap PCR and then connected to the target vector pCXUN‐Cas using the ClonExpress II One Step Cloning Kit (Vazyme, China) (Sun *et al*., 2016).

To construct the overexpression vector, the full‐length cDNA of *OsAGO17* (AK240838, J065016C10) without the termination codon TAG was cloned and inserted into a modified pU2301 vector controlled by the maize ubiquitin promoter and with a 3 × flag tag downstream of the inserted gene (Sun and Zhou, 2008).

A 2‐kb fragment upstream of the translation initiation codon ATG of the *OsAGO17* gene was fused to the GUS reporter gene. The primer sequences are provided in Table S8.

The constructs were transformed into *Agrobacterium* strain *EH105A* and into the callus of ZH11 (Wu *et al*., 2003). To measure the main agronomic characteristics, including plant height, plants were cultivated during the normal rice‐growing season in the experimental farm at Huazhong Agricultural University (Wuhan, China).

### QRT‐PCR analysis

Different tissues were collected and ground in liquid nitrogen. Total RNA (4 μg) was isolated using TRIzol reagent (Invitrogen, Waltham, MA). First‐strand cDNA was reverse transcribed using SuperScript III reverse transcriptase (Invitrogen). QRT‐PCR was performed on an ABI StepOne^TM^ real‐time PCR instrument (Applied Biosystems, Carlsbad, CA). *OsGAPDH* was detected in parallel and used as an internal reference. Each experiment was performed with three biological replicates. The primer sequences are provided in Table S8.

### Cell length observation

The second sheaths of 3‐week‐old seedlings were immersed in 75% ethanol to dissolve chloroplasts, microscopically examined (BX53; Olympus, Tokyo, Japan) and photographed with a CCD camera (SPOT Flex; Diagnostic Instruments, Florida City, FL). The internodes of mature plants were softened with 15% hydrofluoric acid at least two weeks before cutting to 8 μm with a microtome (RM2265; Leica, Wetzlar, Germany). Sections were double stained with annona red and fast green, microscopically examined and photographed with SPOT Flex. To observe glumes, we cut the middle sections of completely dried rice lemmas to obtain small squares, and the surfaces of the squares were coated with gold dust and observed with a scanning electron microscope (JSM‐6390 LV, JEOL, Tokyo, Japan). ImageJ software was used to measure the cell length.

### sRNA sequencing and analysis

Total RNA extracted from 3‐week‐old seedlings of *ago17‐1*, OE1 and ZH11 was separated on 15% agarose gels to extract small RNA (16–30 nt). The qualified libraries were sequenced on an Illumina Hiseq 2500 platform, and 50‐bp single‐end reads were generated. From the generated data, adapter sequences were removed, and reads ranging from 17 to 28 nt in length were selected for further analysis.

Noncoding RNAs were removed based on Rfam (http://rfam.xfam.org/). sRNAs that exhibited perfect mapping to the *O. sativa* genomic sequence (version 7.0) from the MUS database were sorted and used for further study. The sRNA sequences were aligned to sequences of precursor transcript of miRNAs in miRBase (http://www.mirbase.org/) to annotate the miRNAs. The ta‐siRNA database (http://bioinfo.jit.edu.cn/tasiRNADatabase/) and Plant Natural Antisense Transcripts DataBase (http://bis.zju.edu.cn/pnatdb/) were used to predict ta‐siRNA and nat‐siRNA. MiRDeep2 was used to predict novel miRNAs (Friedlander *et al*., 2012). We used DESeq to calculate the change in expression between *ago17‐1* and ZH11 and between OE1 and ZH11 (Anders and Huber, 2010) and deemed the expression of sRNA significantly changed if |log2FC| ≥ 1 and *P*adj < 0.05. psRNAtarget was used to predict miRNA target genes, applying a cut‐off mispairing score of ≤ 5.0, and the range of central mismatch leading to translational inhibition was 9‐11 (Dai and Zhao, 2011).

### Stem‐loop RT‐PCR

Based on the miRNA sequence, specific RT primers and forward primers were designed. The joint sequence of the RT primer was used to design a reverse primer (Chen *et al*., 2005). The RT primer was used instead of oligo‐dT when performing reverse transcription PCR, and the forward primer and universal reverse primer were used for qRT‐PCR. Rice *U6* was detected in parallel and used as an internal reference. The primer sequences are provided in Table S8.

### Western blot analysis

Western blot analyses were performed as previously described (Tan *et al*., 2007). Total protein from the samples was collected and separated on a 12% SDS‐polyacrylamide gel and then transferred to a PVDF membrane (Millipore, MASS., Boston, MA). Western blotting was performed using antibodies against FLAG (F3165; Sigma, St. Louis, MO). SuperSignal^™^ West Pico Chemiluminescent Substrate (Thermo, Waltham, MA) was used to visualize the signal.

### Small RNA detection

Twenty micrograms of total RNA was isolated from different tissues, and low‐molecular‐weight small nuclear RNA was separated as previously described (Diaz‐Pendon *et al*., 2007). Oligonucleotide sequences that were complementary to OsmiR397b with a 5′‐terminal ^[γ‐32P]^ATP label were used as probes. A short sequence of U6 was also used as a probe and served as a reference. Hybridization and subsequent washes were performed as previously described (Diaz‐Pendon *et al*., 2007). Blots were exposed to storage phosphor screens and scanned using a phosphor imager (Molecular Dynamics).

### IP analysis

Immediately after harvesting, 1 g of leaf tissue was pulverized in liquid nitrogen with 1.5 mL of extraction buffer: 20 mm Tris (pH 7.5), 150 mm NaCl, and 1% Triton X‐100, with 1 mm protease inhibitor cocktail (Roche, Basel, Switzerland). The homogenized samples were centrifuged twice at 10 000×***g*** and 4 °C for 15 min. The supernatant was isolated, and 2 μl of anti‐FLAG (F1804; Sigma) was added, and then, the sample was rotated for 2 h at 4 °C. Proteins A/G magnetic agarose beads (78609; Thermo) were washed with TBS, added to the samples and incubated for 2 h at room temperature. The beads were washed six times with 0.1 M glycine solution, and the precipitated material was analysed by Western blot assays. The RNA was isolated, and miRNAs were tested by stem‐loop RT‐PCR.

## Conflict of Interest

The authors declare that they have no conflict of interest.

## Author Contribution

JY conceived and designed this research as well as wrote the manuscript. JZ conducted the experiments and wrote the manuscript. JZ, WH, ZP, HZ and FL performed the experiments. All authors read and approved the manuscript.

## Supporting information


**Figure S1** Protein sequence analysis of OsAGO17.
**Figure S2** Phenotypic observation of RNAi lines.
**Figure S3** The quality of seed in *OsAGO17* transgenic lines and WT.
**Figure S4** Seeds were smaller in *OsAGO17* RNAi lines compared with WT.
**Figure S5** Histological observation in RNAi‐lines.
**Figure S6** Expression analyses of cell expression and cell proliferation genes and starch and lipid‐metabolizing enzyme genes.
**Figure S7** Expression patterns of *OsAGO17* and *LAC* genes during the life cycle of the rice plant.

**Table S1** Analysis of phenotype parameters in *OsAGO17* OE T_1_ lines and WT.
**Table S2** Analysis of phenotype parameters in *ago17* T_1_ lines and WT.
**Table S3** Analysis of phenotype parameters in *OsAGO17* RNAi T_1_ lines and WT.
**Table S4** Analysis of yield parameters among *OsAGO17* OE lines, *ago17* and ZH11.
**Table S5** OsmiRNA expression analysis in ZH11 and *ago17‐1*.
**Table S6** OsmiRNA expression analysis in ZH11 an*d* OE1.
**Table S7** Target genes analysis of different expression OsmiRNA.
**Table S8** Primers used for functional analysis of *OsAGO17*.
