# Supplementary material for: A putative AGO protein, OsAGO17, positively regulates grain size and grain weight through OsmiR397b in rice
Source: Plant Biotechnol J. 2019 Oct 7;18(4):916–28. doi: 10.1111/pbi.13256 (PMC7061870; doi:10.1111/pbi.13256)
Supplement: Supplementary file 2 — Table S1 Analysis of phenotype parameters in OsAGO17 OE T1 lines and WT. Table S2 Analysis of phenotype parameters in ago17 T1 lines and WT. Table S3 Analysis of phenotype parameters in OsAGO17 RNAi T1 lines and WT. Table S4 Analysis of yield parameters among OsAGO17 OE lines, ago17 and ZH11. Table S8 Primers used for functional analysis of OsAGO17. [file PBI-18-916-s003.doc]

|  | Relative expression | Plant height (cm) | Panicle length (cm) | Weight of 1000 seed (g) | Setting rate | Spikelet number per panicle |
| --- | --- | --- | --- | --- | --- | --- |
| OE-WT | 1.02 | 112.5 | 22.15 | 25.69 | 0.85 | 195.6 |
| ZH11 | 1.01 | 112.7 | 24.21 | 25.47 | 0.90 | 201.3 |
| OE11-13 | 2.71 | 109.5 | 23.10 | 27.81 | 0.82 | 162.3 |
| **OE11-23 (OE1)** | 3.55 | 110.2 | 23.10 | 30.41 | 0.71 | 178.0 |
| OE11-27 | 2.82 | 111.4 | 23.53 | 28.07 | 0.64 | 168.7 |
| OE13-3 | 2.73 | 112.7 | 24.77 | 28.56 | 0.77 | 159.3 |
| OE13-5 | 2.91 | 110.2 | 22.93 | 29.78 | 0.73 | 148.0 |
| **OE13-13 (OE2)** | 2.93 | 111.3 | 23.30 | 29.73 | 0.65 | 170.3 |
| **OE19-2 (OE3)** | 3.22 | 107.9 | 23.47 | 30.21 | 0.61 | 159.0 |
| OE19-16 | 3.01 | 109.8 | 24.93 | 29.88 | 0.72 | 167.67 |
| OE19-23 | 2.91 | 109.4 | 23.33 | 28.14 | 0.61 | 141.33 |

**Table S1** Analysis of phenotype parameters in *OsAGO17* OE T_1_ lines and WT.

**Table S2** Analysis of phenotype parameters in *ago17* T_1_ lines and WT.

|  | Relative expression | | Plant height (cm) | Panicle length (cm) | Weight of 1000 seed (g) | Setting rate | Spikelet number per panicle |
| --- | --- | --- | --- | --- | --- | --- | --- |
| WT | 1.01 | 114.32 | | 24.85 | 25.81 | 0.89 | 201.40 |
| ZH11 | 1.02 | 115.02 | | 24.65 | 25.54 | 0.92 | 189.53 |
| **C17-3 ( *ago17-1*)** | 0.13 | 88.43 | | 22.88 | 21.36 | 0.70 | 149.40 |
| **C19-2 (*ago17-2*)** | 0.35 | 89.51 | | 22.92 | 21.73 | 0.69 | 170.00 |

**Table S3 Analysis of phenotype parameters in *OsAGO17* RNAi T_1_ lines and WT.**

|  | Relative expression | Plant height (cm) | Panicle length (cm) | | Weight of 1000 seed (g) | Setting rate | Spikelet number per panicle |
| --- | --- | --- | --- | --- | --- | --- | --- |
| Ri-WT | 1.02 | 117.1 | | 25.01 | 25.58 | 0.87 | 185.8 |
| ZH11 | 1.00 | 116.9 | | 24.71 | 25.57 | 0.91 | 188.3 |
| R3-3 | 0.42 | 99.1 | | 21.80 | 23.12 | 0.82 | 156.3 |
| **R3-13 (R1)** | 0.35 | 96.9 | | 22.91 | 22.37 | 0.83 | 160.2 |
| R3-11 | 0.51 | 102.1 | | 22.96 | 23.15 | 0.69 | 169.0 |
| R8-16 | 0.38 | 95.9 | | 22.76 | 22.69 | 0.73 | 173.0 |
| R8-5 | 0.43 | 98.1 | | 22.06 | 22.72 | 0.62 | 165.0 |
| **R8-22 (R2)** | 0.28 | 88.9 | | 23.5 | 21.83 | 0.79 | 169.3 |
| R15-5 | 0.54 | 103.9 | | 22.36 | 23.37 | 0.85 | 165.0 |
| **R15-7 (R3)** | 0.44 | 100.1 | | 22.30 | 22.64 | 0.84 | 161.3 |
| R15-14 | 0.52 | 102.1 | | 23.50 | 23.36 | 0.76 | 168.0 |

**Table S4 Analysis of yield parameters among *OsAGO17* OE lines, *ago17* and ZH11.**

|  | Panicle length (cm) | Number of primary branches | Spikelet number per panicle | Setting rate (%) | No. of tillers per plant | grain yield per plant |
| --- | --- | --- | --- | --- | --- | --- |
| ZH11 | 23.65 ± 0.37 | 11.9 ± 1.46 | 151.6 ± 16.5 | 87.21 ± 0.53 | 11.23 ± 1.23 | 17.97 ± 1.02 |
| *ago17-1* | 20.40 ± 0.52** | 11.6 ± 2.73 | 95.7± 18.1** | 72.63 ± 2.57** | 11.68 ± 1.31 | 9.83 ± 2.15** |
| *ago17-2* | 20.71 ± 0.41** | 11.59 ± 2.35 | 96.8 ± 15.3** | 73.88 ± 2.63** | 11.96 ± 1.29 | 9.69 ± 2.07** |
| OE1 | 24.92 ± 0.62** | 11.04 ± 1.98 | 115.4 ± 16.5** | 84.51 ± 2.95 | 10.98 ± 1.28 | 16.78 ± 2.12 |
| OE2 | 24.35 ± 0.47* | 11.18 ± 2.31 | 117.3 ± 16.1** | 83.11 ± 3.64 | 11.35 ± 1.35 | 17.2 ± 2.29 |
| OE3 | 24.63 ± 0.51** | 11.3 ± 2.21 | 113.9 ± 15.3** | 82.98 ± 3.12 | 11.26 ± 1.19 | 17.13 ± 3.94 |

Data are presented as mean ± SE, ** P＜ 0.01, * P＜ 0.05, by student’s *t*-test, n = 30.

**Table S5** OsmiRNA expression analysis in ZH11 and *ago17-1*.

**Table S6** OsmiRNA expression analysis in ZH11 an*d* OE1.

**Table S7** Target genes analysis of different expression OsmiRNA.

**Table S8** Primers used for functional analysis of *OsAGO17*.

| Name | Forward primer (5'-3') | | Reverse primer (5'-3') |  |
| --- | --- | --- | --- | --- |
| **Primer for RNAi fragment amplification** | | | |  |
| RAGO17 | GCGGTACCACTAGTTCTTGGCTGGTTCTTTC | | GCGGATCCGAGCTCACCTGAATCTCGCTGAA |  |
| **Primer for overexpression fragment amplification** | | | |  |
| OEAGO17 | ACGATAGCCGGTACCATGGAGAGTCAGAGAATGACCTGGCTC | | TTTGTAATCGGATCCGCAGAAAAACATGGCCCCTTTCAAAT |  |
| **Primers for CRISPR-Cas9 vector construction** | | | |  |
| U3AGO17 | ATGGTGGGAGAGTGAAAAGCGTTTTAGAGCTAGAAATAGCAAGTTA | | GCTTTTCACTCTCCCACCATGCCACGGATCATCTGCACAAC |  |
| U3 | CCCCTTTCGCCAGGGGTACCGTAATTCATCCAGGTCTCCAAG | | TACGAATTCGAGCTCGGTACCGCTGTGCCGTACGACGGTACG |  |
| pCXUNF | | TGTGTGGAATTGTGAGCGGATA | GTTTTACAACGTCGTGACTGGG |  |
| **Primers for subcellular localization vector** | | | |  |
| SLAGO17 | | TCTATCGATTCTAGAATGGAGAGTCAGAGAATGACCTGGCTC | CACCATGGCTCTAGAGCAGAAAAACATGGCCCCTTTCAAAT |  |
| **Primer for CRISPR plants detection** | | | |  |
| crAGO17 | | TGCATGTGTTATGCTGTAGGTCTG | GTGCAGATTCTACACATCCAGGTT |  |
| **Primers for promoter vector** | | | |  |
| AGO17-GUS | | AACTGCAGTATATGTGTAAAACAGAGGATTG | CGGGATCCGATTTCGTAATCTAATCGGT |  |
| **Primers for qRT-PCR** | | | |  |
| *OsAGO17* | | TCAGAAGGTATGATTGGCGAAA | ACCCAAACCAGAATCTTTCTGC |  |
| *GAPDH* | | GGATATGTTGAGGAAGACCTGGTTTC | CTACTGGGTCTTGGCCATGTGG |  |
| *OsLAC* | | GAGGAGGTGCCCATCATGTTC | CCTTCAGCTTAAACGTGTCTTGG |  |
| *OsLAC14* | | AACACCGTCGGCGTGCCCGCCG | TTAAGTTGGTGCAAATGTGGAA |  |
| *OsLAC29* | | GGGTTTGGTTCATGCACTGCCA | CTAGCACTGTGGCAAGTCCGAT |  |
| *OsLAC8* | | AGGCATGTGGTTCTTCCATT | CTAGCACTTCGGCAGATCGG |  |
| *PGL1* | | GCATCAGGAGGTCGACGACCTGA | CTACATCAGAAGGCTGCGGATGA |  |
| *PGL2* | | ATGTCGAGCAGAAGGTCGTC | TCCCGGTGAAGGCTCTTGATGT |  |
| *APG* | | CTTTCGAGCTCGCAGAGGAG | CCATCGACATCATCTGCACC |  |
| *GS5* | | CAGGTTGCTGGGAGGTTTGT | CTCACCATGAAGGAATGCAT |  |
| *GSN1* | | GGTGAATATGAGAGCAGCAA | CTCCGTTTCATGCTCTTTGA |  |
| *GW2* | | ACCATGCCGCAGCTGCCTTT | GCCAACCCTTGCGAGTGTGT |  |
| *GW7* | | AAAGAATGCTATTGGTTGAG | CATATGACCTCGTCAATGAG |  |
| *SRS3* | | TTCGTCCAAAACCTCCCACA | CTCCACGAAAGCAATCCCAG |  |
| *OsD2* | | CTGACGGAGCTGATGGGCAA | TGGGAGGACTTGAAGAAGGCGC |  |
| *OsBRI1* | | TCGGTGACTGCCAGAGCTTGGT | CCGTCCAACTATGAGGCCAACATT |  |
| *OsGSK2* | | GCTGGGTGTGTTCTTGCAGAGC | TTCCTCACGGGTTGGTGTACCA |  |
| *OsGA3ox2* | | ACGAGTTGCTGAGGTTGT | GGTACCAGTTGAGGTGCA |  |
| *OsGA2ox3* | | ACCATTGCCACAGCTGCTGGGG | CTACTTCTTCTCAAACTGGGCC |  |
| *OsCPS1* | | AGGGATGCCTGGGCTGGATT | CGGTCTGCATGAGAGCATAAGCA |  |
| *OsGA3ox1* | | TCCGGTACCCGAAGCAGATGT | AGTCGTACTCCTCCATGGCGGA |  |
| *Osbrd1* | | GGGAAGGAAGTAGGCACCGTAGAA | TGTAACCCGTTGGGAGCTTCAA |  |
| **Primers for stem loop qRT-PCR** | | | |  |
|  | | qRT-PCR F | Stem-loop RT primer |  |
| Osa-miR397b | | gcgaTCATTGAGTGCAGCG | GTCGTATCCAGTGCAGGGTCCGAGGTATTCGCACTGGATACGACcatcaac |  |
| Osa-miR398b | | gcgaTGTGTTCTCAGGTCG | GTCGTATCCAGTGCAGGGTCCGAGGTATTCGCACTGGATACGACcaggggc |  |
| Osa-miR169b | | gcgaCAGCCAAGGATGAC | GTCGTATCCAGTGCAGGGTCCGAGGTATTCGCACTGGATACGACccggcaa |  |
| Osa-miR169r-3p | | gcgaTGGCAAGTCTCCTC | GTCGTATCCAGTGCAGGGTCCGAGGTATTCGCACTGGATACGACggtagcc |  |
| Osa-miR1859 | | gcgaTTTCCTATGACGTCC | GTCGTATCCAGTGCAGGGTCCGAGGTATTCGCACTGGATACGACttggaat |  |
| Osa-miR2837a | | gcgaAAGTTTGGACTTAAAT | GTCGTATCCAGTGCAGGGTCCGAGGTATTCGCACTGGATACGACgttacca |  |
| miRUniverseR | | CCAGTGCAGGGTCCGAGGT |  |  |
| U6 | | TACAGATAAGATTAGCATGGCCCC | GGACCATTTCTCGATTTGTACGTG |  |
| **Primers for northern blot** | | | |  |
| Osa-miR397b | | GGTTCATCAACGCTGCACTCAA |  |  |
| U6 | | TGTATCGTTCCAATTTTATCGGATGT |  |  |
