# Supplementary material for: A putative AGO protein, OsAGO17, positively regulates grain size and grain weight through OsmiR397b in rice
Source: Plant Biotechnol J. 2019 Oct 7;18(4):916–28. doi: 10.1111/pbi.13256 (PMC7061870; doi:10.1111/pbi.13256)
Supplement: Supplementary file 1 — Figure S1 Protein sequence analysis of OsAGO17. Figure S2 Phenotypic observation of RNAi lines. Figure S3 The quality of seed in OsAGO17 transgenic lines and WT. Figure S4 Seeds were smaller in OsAGO17 RNAi lines compared with WT. Figure S5 Histological observation in RNAi‐lines. Figure S6 Expression analyses of cell expression and cell proliferation genes and starch and lipid‐metabolizing enzyme genes. Figure S7 Expression patterns of OsAGO17 and LAC genes during the life cycle of the rice plant. [file PBI-18-916-s004.docx]

**Figure S1** Protein sequence analysis of OsAGO17. N terminal (A) and Part of PIWI domains (B) of some proteins.

**Figure S2** Phenotypic observation of RNAi lines. Relative expression levels of OsAGO17 (A) and OsAGO1s (B) in RNAi lines. C Grain of RNAi lines and WT. Bar = 1 cm. D-I. Length (C) and width of ten seeds (D), weight of 1,000 seeds (E), panicle Length (G), spikelet number per panicle (H), and setting rate (I) of RNAi lines and WT. J. Plant height of RNAi lines and WT. Bar = 10 cm. K-M. Pant height at mutual period (K) and at different days after germination (L), different internode lengths (M) of RNAi lines and WT. The data are presented as mean ± SE, n = 30, ** P＜ 0.01, * P＜ 0.05, by Student’s *t*-test.

**Figure S3** The quality of seed in *OsAGO17* transgenic lines and WT. A-B. The seed of *ago17* and OE-lines were with on obvious chalkiness. Bar = 1 mm. C-D. The content of total proteins (C) and starch (D) of seed. The data are presented as mean ± SE, n = 3, by Student’s *t*-test.

**Figure S4** Seeds were smaller in *OsAGO17* RNAi lines compared with WT. The seed at 3 (A), 5 (B), 7 (C), 10 (D) and 12 (E) DAP seed of RNAi lines and WT. Bar = 2 mm. The 3 (F) and 5 (G) DAP seed in the ZH11, *ago17* and OE lines. Bar = 1 mm.

**Figure S5** Histological observation in RNAi-lines. A. Longitudinal section of first internode of stem at mature period, bar =100 μm. B: Cell length of stem cortex cell. The data are presented as mean ± SE, ** P ＜ 0.01, by Student’s *t*-test. n = 15 sections. C. Epidermal layers of mature seed glume middle section, bar = 100 μm.

**Figure S6** Expression analyses of cell expression and cell proliferation genes and starch and lipid-metabolizing enzyme genes by quantitative RT-PCR.

**Figure S7** Expression patterns of *OsAGO17* and *LAC* genes during the life cycle of the rice plant.
